# ^47^Sc as useful β^–^-emitter for the radiotheragnostic paradigm: a comparative study of feasible production routes

**DOI:** 10.1186/s41181-017-0024-x

**Published:** 2017-06-02

**Authors:** Katharina A. Domnanich, Cristina Müller, Martina Benešová, Rugard Dressler, Stephanie Haller, Ulli Köster, Bernard Ponsard, Roger Schibli, Andreas Türler, Nicholas P. van der Meulen

**Affiliations:** 1 Laboratory of Radiochemistry, Paul Scherrer Institut, 5232 Villigen-PSI, Switzerland; 2 Department of Chemistry and Biochemistry University of Bern, 3012 Bern, Switzerland; 3 Center for Radiopharmaceutical Sciences ETH-PSI-USZ, Paul Scherrer Institut, 5232 Villigen-PSI, Switzerland; 4 Department of Chemistry and Applied Biosciences, ETH Zurich, 8093 Zurich, Switzerland; 5 Institut Laue-Langevin, 38000 Grenoble, France; 6 SCK.CEN, BR2 Reactor, 2400 Mol, Belgium

**Keywords:** ^47^Sc, Matched pairs, Theragnostics, Radionuclide production, ^46^Ca, ^47^Ti, Thermal and fast neutrons, SPECT/CT imaging

## Abstract

**Background:**

Radiotheragnostics makes use of the same molecular targeting vectors, labeled either with a diagnostic or therapeutic radionuclide, ideally of the same chemical element. The matched pair of scandium radionuclides, ^44^Sc and ^47^Sc, satisfies the desired physical aspects for PET imaging and radionuclide therapy, respectively. While the production and application of ^44^Sc was extensively studied, ^47^Sc is still in its infancy. The aim of the present study was, therefore, to investigate and compare two different methods of ^47^Sc production, based on the neutron irradiation of enriched ^46^Ca and ^47^Ti targets, respectively.

**Methods:**

^47^Sc was produced by thermal neutron irradiation of enriched ^46^Ca targets via the ^46^Ca(n,γ)^47^Ca → ^47^Sc nuclear reaction and by fast neutron irradiation of ^47^Ti targets via the ^47^Ti(n,p)^47^Sc nuclear reaction, respectively. The product was compared with regard to yield and radionuclidic purity. The chemical separation of ^47^Sc was optimized in order to obtain a product of sufficient quality determined by labeling experiments using DOTANOC. Finally, preclinical SPECT/CT experiments were performed in tumor-bearing mice and compared with the PET image of the ^44^Sc labeled counterpart.

**Results:**

Up to 2 GBq ^47^Sc was produced by thermal neutron irradiation of enriched ^46^Ca targets. The optimized chemical isolation of ^47^Sc from the target material allowed formulation of up to 1.5 GBq ^47^Sc with high radionuclidic purity (>99.99%) in a small volume (~700 μL) useful for labeling purposes. Three consecutive separations were possible by isolating the in-grown ^47^Sc from the ^46/47^Ca-containing fraction. ^47^Sc produced by fast neutron irradiated ^47^Ti targets resulted in a reduced radionuclidic purity (99.95–88.5%). The chemical purity of the separated ^47^Sc was determined by radiolabeling experiments using DOTANOC achievable at specific activities of 10 MBq/nmol. In vivo the ^47^Sc-DOTANOC performed equal to ^44^Sc-DOTANOC as determined by nuclear imaging.

**Conclusion:**

The production of ^47^Sc via the ^46^Ca(n,γ)^47^Ca nuclear reaction demonstrated significant advantages over the ^47^Ti production route, as it provided higher quantities of a radionuclidically pure product. The subsequent decay of ^47^Ca enabled the repeated separation of the ^47^Sc daughter nuclide from the ^47^Ca parent nuclide. Based on the results obtained from this work, ^47^Sc shows potential to be produced in suitable quality for clinical application.

**Electronic supplementary material:**

The online version of this article (doi:10.1186/s41181-017-0024-x) contains supplementary material, which is available to authorized users.

## Background

Over the past few years, the concept of personalized medicine, where patient treatment is performed according to an individually tailored treatment regime, has gained much recognition (Kraeber-Bodere and Barbet 2014). In nuclear medicine, this approach is realized by exploiting diagnostic techniques, such as non-invasive imaging by means of Positron Emission Tomography (PET) and Single Photon Emission Computed Tomography (SPECT), together with individualized radiotherapeutic treatment (Velikyan 2012). The resulting combination became known as the theragnostic approach and comprises the use of the same molecular targeting vectors, labeled either with a diagnostic or therapeutic radionuclide (Baum and Kulkarni 2012). Ideally, the employed radionuclides represent a matched pair, where both are radioisotopes of the same chemical element. Only a limited number of matching radionuclides entail suitable decay characteristics for radiotheragnostic application (Rösch and Baum 2011); of those the radionuclides of scandium, ^44^Sc/^43^Sc and ^47^Sc, are interesting candidates. Based on the physical and chemical characteristics, the β^−^-emitter ^47^Sc is particularly interesting for radionuclide therapy, while the decay characteristics of ^44^Sc and ^43^Sc are well-suited for diagnostic PET imaging (Table 1) (Rösch 2012; Müller et al. 2014a, 2014b; Walczak et al. 2015).

**Table 1:** Nuclear data of theragnostic radionuclides for therapy and PET imaging

Therapeutic radionuclide				Diagnostic radionuclide (positron emitter)			
	Half-life [d]	Eβ^−^_av_ [keV]	Eγ [keV] (Iγ [%])		Half-life [h]	Eβ^+^_av_ [keV] (I[%])	Eγ [keV] (Iγ [%])
^177^Lu	6.65	134	113 (6.4) 208 (11.0)	^68^Ga	1.13	830 (89)	1077 (3.0)
^47^Sc	3.35	162	159 (68.3)	^44^Sc	4.04	632 (94)	1157 (99.9)
				^43^Sc	3.89	476 (88)	372 (23.0)

Intensities less than 5% were not considered

This matched pair would present an attractive alternative to ^68^Ga and ^177^Lu, which are currently used in clinics for PET imaging and therapy, respectively (Oh et al. 2011). Ga(III) and Lu(III) can be coordinated by 1,4,7,10-tetraazacyclododecane-1,4,7,10-tetraacetic acid (DOTA)-complexes (Majkowska-Pilip and Bilewicz 2011), however, they do not share the same coordination chemistry. Lu(III) is coordinated by all carboxyl groups of the octadentate DOTA (Viola-Villegas and Doyle 2009; Parus et al. 2015), while Ga(III) has a preference for the coordination number six, leaving two uncoordinated carboxyl groups in the Ga-DOTA-complex (Viola-Villegas and Doyle 2009; Majkowska-Pilip and Bilewicz 2011). As a result, these structural differences may have an influence on the radioconjugate’s chemical properties and, consequently, on the in vivo kinetics and receptor binding affinity (Reubi et al. 2000; Majkowska-Pilip and Bilewicz 2011). By using chemically identical radionuclides such as ^44^Sc/^47^Sc–known to form stable complexes with DOTA–this limitation could be addressed.

The physical half-life of ^44^Sc of 3.97 h (recently re-determined as 4.04 h (Garcia-Torano et al. 2016), is almost 4-fold longer than that of ^68^Ga (T_1/2_ = 68 min) and, hence, allows its use with biomolecules with slower kinetics. Due to the possibility of shipping ^44^Sc-radiopharmaceuticals over long distances, it can also facilitate logistics as it would allow centralized production and distribution to remote hospitals (Chakravarty et al. 2014; van der Meulen et al. 2015). The increased availability of ^44^Sc has initiated a number of preclinical in vitro and in vivo studies with DOTA-conjugated biomolecules (Müller et al. 2013a, 2013b; Hernandez et al. 2014) and, recently, labeling of NODAGA (1,4,7-triazacyclononane,1-glutaric acid-4,7-acetic acid)-functionalized peptides and DTPA (N-[(R)-2-amino-3-(para-isothiocyanato-phenyl)propyl]trans-(S,S)-cyclohexane-1,2-diamine N,N,N’,N”N”-pentaacetic acid)-functionalized antibodies was also demonstrated (Chakravarty et al. 2014; Domnanich et al. 2016).

The emission of low-energy β^−^-particles from ^47^Sc (Eβ^−^_av_ = 162 keV, Table 1) is particularly interesting for targeted radionuclide therapy of small tumors and cancer metastases, similar to the clinically-established ^177^Lu (Eβ^−^_av_ = 134 keV, T_1/2_ = 6.65 d, Table 1). Moreover, the shorter half-life of ^47^Sc (T_1/2_ = 3.35 d) would encourage its use, in conjunction with small molecules, with relatively fast pharmacokinetic profiles. In analogy to ^177^Lu, the decay of ^47^Sc is characterized by the co-emission of γ-rays with an ideal energy (Eγ = 159 keV, Table 1) for SPECT imaging (Müller et al. 2014a).

**Fig. 1:**
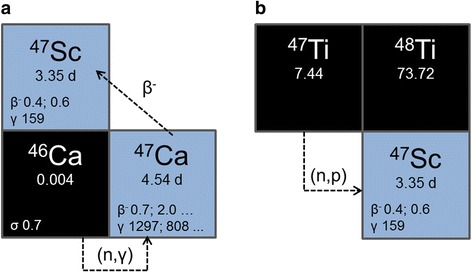
Nuclear reactions for production of ^47^Sc from ^46^Ca via ^46^Ca(n,γ)^47^Ca → ^47^Sc (**a**) and from ^47^Ti via ^47^Ti(n,p)^47^Sc (**b**)

The availability of high ^47^Sc activity with adequate purity becomes a crucial issue for the realization of more detailed preclinical investigations and future clinical applications. So far, successful production of ^47^Sc was described by two different neutron induced reactions: ^47^Ti(n,p)^47^Sc and ^46^Ca(n,γ)^47^Ca → ^47^Sc (Fig. 1) (Bartoś et al. 2012; Müller et al. 2014a). To produce ^47^Sc from ^47^Ti, fast neutrons (E_n_ > 1 MeV) are required, while the ^46^Ca(n,γ)^47^Ca reaction is induced by thermal neutrons (E_n_ = 0.025 eV) (Bartoś et al. 2012). Proton irradiation of enriched ^48^Ti targets made ^47^Sc available via the ^48^Ti(p,2p)^47^Sc nuclear reaction, however, too much of the long-lived ^46^Sc was co-produced (Srivastava 2012). An alternative ^47^Sc production route considers photonuclear reactions on enriched ^48^Ti and ^48^Ca targets, respectively (Yagi and Kondo 1977; Mamtimin et al. 2015; Rane et al. 2015; Starovoitova et al. 2015). So far only the former route was studied in detail with enriched targets (Yagi and Kondo 1977), while for the latter only natural target material was used for initial benchmark experiments.

The aim of the present study was to optimize the parameters of the previously-reported production process for ^47^Sc from enriched ^46^Ca targets (Müller et al. 2014a) in order to reproducibly obtain increased ^47^Sc yields in a formulation allowing direct preclinical application after radiolabeling and to compare it with the method using ^47^Ti as target material. ^47^Sc labeling experiments with DOTANOC were performed as part of the quality control. In the second part of the study the stability of ^47^Sc-labeled DOTANOC was investigated and SPECT/CT imaging studies were performed in tumor-bearing mice to compare the performance of ^47^Sc-DOTANOC with the previously obtained PET images of the ^44^Sc-labeled counterpart. Moreover, comparison of SPECT images obtained with mice injected with ^177^Lu-DOTANOC were also performed.

## Methods

### Chemicals

Enriched ^46^CaCO_3_ (83.09% ^40^Ca, 1.19% ^42^Ca, 0.36% ^43^Ca, 8.55% ^44^Ca, 5.00 ± 0.50% ^46^Ca, 1.81% ^48^Ca, Trace Sciences International, USA) was used as target material for thermal neutron irradiation. Enriched ^47^TiO_2_ (0.41% ^46^Ti, 95.7 ± 0.3% ^47^Ti, 3.61% ^48^Ti, 0.15% ^49^Ti, 0.13% ^50^Ti, Isoflex, USA) was reduced to ^47^Ti metal and used as target material for fast neutron irradiation. Prior to irradiation, a precursory scan for trace metals by ICP-OES (Perkin Elmer Optima 3000) was performed.

The chemical separation of Sc from Ca was performed on a *N,N,N’,N’*-tetra-n-octyldiglycolamide, non-branched resin (DGA, particle size 50–100 μm, TrisKem International, France). SCX cation exchange cartridges (100 mg Bond Elut SCX, particle size 40 μm, Agilent Technologies Inc., USA) or DGA extraction chromatographic resin were used for the preconcentration of Sc. Chemical separations were performed with MilliQ water, hydrochloric acid (HCl, 30% Suprapur, Merck KGaA, Germany) and sodium chloride (NaCl, Trace Select, ≥99.999%, Fluka Analytical, Germany). For the ^47^TiO_2_ reduction process, calcium hydride (CaH_2_, metals basis, Mg <1%, Alfa Aesar, Germany), argon (Ar, 99.9999%, Linde, Germany) and acetic acid (CH_3_COOH, 100% Suprapur, Merck KGaA, Germany) were used. Nitric acid (HNO_3_, 65% Suprapur, Merck KGaA, Germany) was required for the preparation of the ^46^Ca targets. DOTANOC acetate was obtained from ABX GmbH, advanced biochemical compounds, Germany.

### Production of ^47^Sc from enriched ^47^Ti

The reduction of ^47^TiO_2_ was performed at Helmholtz Center for Heavy Ion Research (GSI) in Darmstadt as described elsewhere (Lommel et al. 2014). Briefly, the enriched ^47^TiO_2_ was combined with 40% surplus of calcium hydride and the reduction process was performed under constant argon flow at 900 °C for 1 h. Dilute acetic acid was used for the isolation of the reduced ^47^Ti metal from the co-produced calcium oxide.

To prepare the targets, 0.6–19.9 mg reduced ^47^Ti powder was placed in a quartz glass ampoule (Suprasil, Heraeus, Germany) and sealed. The targets were irradiated with neutrons at the spallation-induced neutron source, SINQ, at Paul Scherrer Institut (PSI) at a fast neutron flux (>1 MeV) of 3.3–3.5 × 10^11^ n cm^−2^ s^−1^ for 1.5–18.9 days and in the BR2 reactor at SCK.CEN, Mol, Belgium in a reflector channel at a fast neutron flux (>1 MeV) of 5.7 × 10^13^ n cm^−2^ s^−1^ for 7 days. ^47^Sc was formed via the ^47^Ti(n,p)^47^Sc nuclear reaction with fast neutrons.

### Production of ^47^Sc from enriched ^46^Ca

To prepare the targets, 65–91 mg enriched ^46^CaCO_3_ powder was dissolved in concentrated nitric acid and evaporated to complete dryness at 60–70 °C. The ^46^Ca(NO_3_)_2_ residue was taken up in dilute nitric acid (~1 M HNO_3_) and an aliquot of the aqueous solution (0.14–0.35 mg ^46^Ca) transferred into a quartz glass ampoule, evaporated to dryness and sealed.

^47^Sc was produced by the irradiation of the described ^46^Ca targets with thermal neutrons at the high flux reactor of Institut Laue-Langevin (ILL) in Grenoble, France at a thermal neutron flux of 1.0–1.4 × 10^15^ n cm^−2^ s^−1^, for 4 to 11 days, and at the BR2 reactor at SCK.CEN, Mol, Belgium at a thermal neutron flux of 3.2 × 10^14^ n cm^−2^ s^−1^ for 7 days, respectively. ^47^Sc was generated by the decay of the formed ^47^Ca (T_1/2_ = 4.54 d) occurring during the irradiation, but also after removal of the ampoule from the reactor.

### Separation of ^47^Sc from ^46^Ca and ^47^Ca

**Fig. 2:**
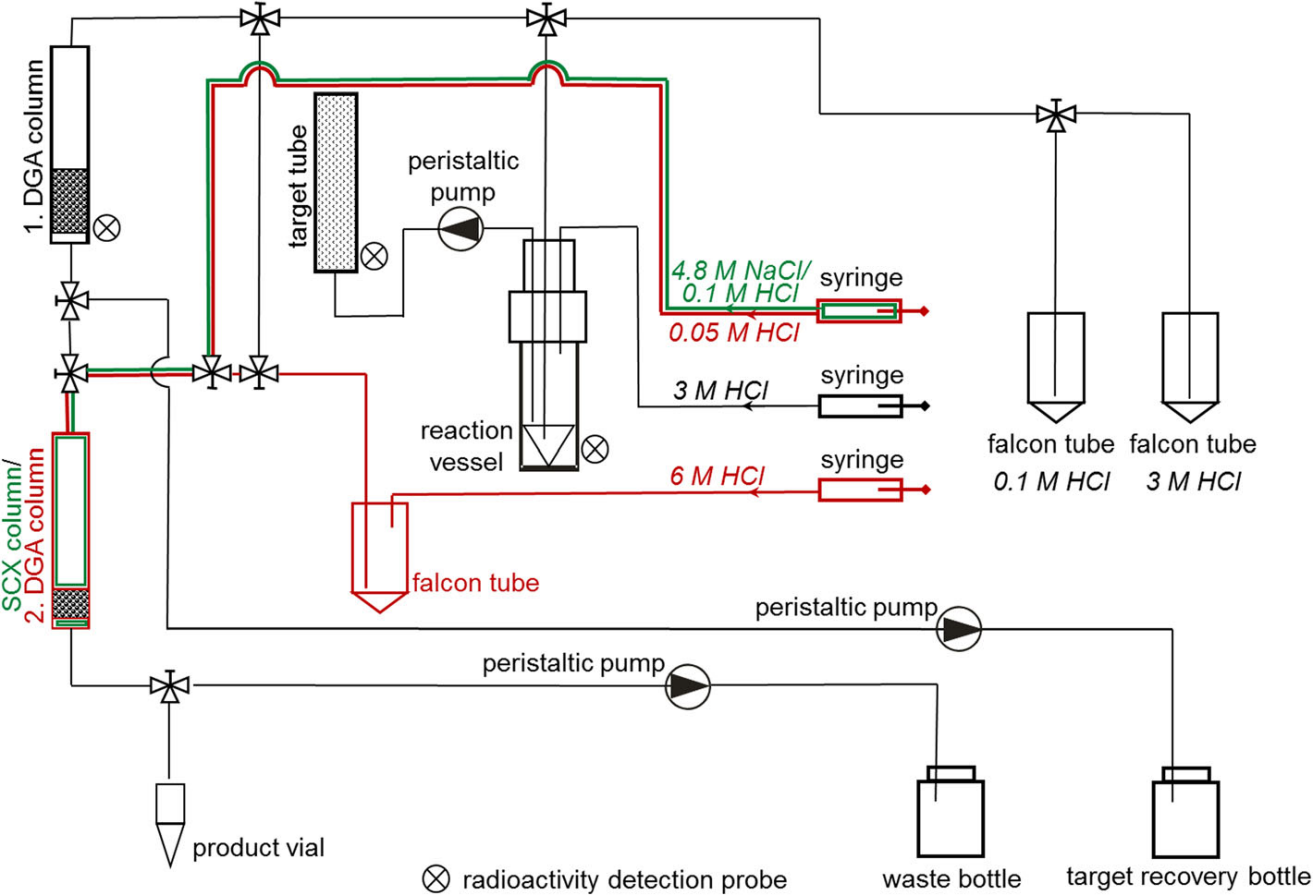
Schematic diagram of the ^47^Sc production panel. The components drawn in *green* are used only to perform separations according to Method A, the parts required for Method B are shown in *red*, while *black* indicates the apparatus components used for both methods

The irradiated ^46^Ca ampoules were delivered to PSI several days post-irradiation (2.6–12.4 d) and the ^47^Sc separation was performed immediately, similarly to previously reported (Müller et al. 2014a). Each ampoule was transferred into a hot cell and the glass surface was cleaned twice with ~20 mL 1.0 M HCl and rinsed twice with ~20 mL MilliQ water. The crushing of the quartz glass ampoule was performed within a plastic target tube in a separate hot cell. Subsequently, the target tube containing the crushed ampoule was attached to the separation panel with the aid of manipulators. The design of the separation panel, including the adaptation of its operation inside the hot cell, was a crucial part of the method development (Fig. 2). The ^46^Ca(NO_3_)_2_ (~10–25 mg) from the ampoule was dissolved in 4 mL 3.0 M HCl and transferred from the target tube to the reaction vessel. A system of syringes, peristaltic pumps and three-way valves (see schematic of the panel in Fig. 2) was used to transfer the reagents from outside into the hot cell. To ensure complete dissolution of the target material, the solution was pumped from the target tube to the reaction vial and back several times. The solution was loaded on a pre-conditioned DGA column (1 mL cartridge filled with 50–70 mg of DGA resin). A second rinse cycle of the crushed glass ampoule with 2.5 mL 3.0 M HCl ensured collection of final traces of the ^47^Sc activity, which were subsequently sorbed onto the DGA resin column. Radioactivity detection probes were attached in the vicinity of the target tube and the DGA column to follow the transfer of the ^47^Sc radioactivity. Further application of 2 mL 3.0 M HCl removed the stable ^46^Ca and radioactive ^47^Ca from the DGA resin. The entire Ca-containing effluent was collected in a separate vessel and kept for consecutive separation of further in-growing ^47^Sc from the decaying ^47^Ca. The sorbed ^47^Sc was eluted from the resin column with 4 mL 0.1 M HCl and sorbed on a second column containing SCX cation exchange resin (Method A). Alternately, the ^47^Sc-containing eluate was collected, acidified to yield a 3.0 M HCl solution and sorbed on a second, smaller DGA resin column (1 mL cartridge filled with 20–25 mg DGA resin) at a slow flow rate of ~0.3 mL/min (Method B), as described by Domnanich et al. (Domnanich et al. 2016). The elution of ^47^Sc from the second column was performed with 700 μL 4.8 M NaCl/0.1 M HCl (for Method A) and with 1.7 mL 0.05 M HCl (for Method B) via a separate valve. In order to collect ^47^Sc in a small volume, the 0.05 M HCl (Method B) was fractionized into three Eppendorf vials; the first contained ~700 μL and the other two ~500 μL each. Fractionized collection was not necessary for Method A, as the highest proportion of the ^47^Sc radioactivity was trapped in a low quantity of eluate.

The renewed generation of ^47^Sc, by the decay of radioactive ^47^Ca in the Ca-containing fraction (^47^Ca and stable ^46^Ca), enabled subsequent separations after a minimum in-growth time of 3 days.

### Radionuclidic purity

To identify the nuclide inventory of the samples, γ-ray spectrometry with an N-type high-purity germanium (HPGe) coaxial detector (EURISYS MESURES, France) and the Ortec InterWinner 7.1 software were employed. The aliquot of ^47^Sc eluate was in the range of 3–15 MBq, while the entire neutron irradiated glass ampoules containing the ^47^Ti were used for the measurements. The counting time was determined by ensuring the measurement error was <4%. To determine small activities of long-lived radionuclidic impurities, γ-spectrometry measurements of the same samples were performed with an extensive counting time several days post-irradiation.

### Radiolabeling for quality control of the produced ^47^Sc

After quantitative determination of the ^47^Sc activity in the eluate with a dose calibrator (ISOMED 2010, Nuclear-Medizintechnik Dresden, GmbH, Germany), the required activity for radiolabeling in 0.05 M HCl was withdrawn from the product vial and 0.5 M sodium acetate solution (pH 8) was added to the ^47^Sc eluate to obtain a pH value of ~4.5. DOTANOC (0.7 mM solution in MilliQ water) was added to the ^47^Sc solution (~50 MBq) to obtain a specific activity of 10–25 MBq/nmol (2–5 nmol DOTANOC). The reaction mixture was incubated at 95 °C for 15 min. The preparation of ^177^Lu-DOTANOC (25 MBq/nmol) was carried out under standard labeling conditions (pH 4.5, 95 °C) using no carrier-added ^177^Lu (purchased from Isotope Technologies Garching GmbH, Germany) (Müller et al. 2013a).

High-performance liquid chromatography (HPLC, Merck Hitachi, LaChrom) with a C-18 reversed-phase column (Xterra^TM^ MS, C18, 5 μm, 150 × 4.6 mm; Waters) was used for determination of the radiolabeled fraction of DOTANOC. The detection was performed with a UV (LaChrom L-7400) and radiodetector (Berthold, HPLC Radioactivity Monitor, LB 506B). The mobile phase consisted of MilliQ water containing 0.1% trifluoracetic acid (A) and acetonitrile (B) with a gradient of 95% A and 5% B to 20% A and 80% B, over a period of 15 min, at a flow rate of 1.0 mL/min.

### In vitro stability of ^47^Sc- and ^177^Lu-labeled DOTANOC

The in vitro stability of ^47^Sc- and ^177^Lu-labeled DOTANOC (radiochemical purities >95%) was investigated in phosphate buffered saline (PBS, pH 7.4). An activity of 50 MBq of ^47^Sc- or ^177^Lu-DOTANOC was diluted with PBS (pH 7.4) to a total volume of 500 μL and incubated at room temperature for 3 days. Once every 24 h an aliquot was withdrawn to determine the integrity of the labeled compound by means of HPLC.

### SPECT/CT imaging with ^47^Sc- and ^177^Lu-DOTANOC

In vivo experiments were approved by the local veterinarian department and conducted in accordance with the Swiss law of animal protection. Female, athymic nude mice (CD-1 nude) at the age of 5–6 weeks were obtained from Charles River Laboratories, Sulzfeld, Germany. AR42J cells (rat exocrine pancreatic tumor cells, European Collection of Cell Cultures ECACC, Salisbury, U.K.) were suspended in PBS (5 × 10^6^ cells in 100 μL) and subcutaneously inoculated on each shoulder. SPECT/CT experiments were performed about 2 weeks after tumor cell inoculation, when the tumor reached a size of about 400 mm^3^.

Imaging studies were performed using a small-animal SPECT camera (NanoSPECT/CT™, Mediso Medical Imaging Systems, Budapest, Hungary) as previously reported (Müller et al. 2014b). The energy peaks for the camera were set at 159.4 keV (± 10%) for the scans with ^47^Sc and 56.1 keV (± 10%), 112.9 keV (± 10%) and 208.4 keV (± 10%) for the scans with ^177^Lu. SPECT/CT scans were followed by CT scans. The images were acquired using Nucline Software (version 1.02, Bioscan Inc., Poway, California, US). The reconstruction was performed iteratively with HiSPECT software (version 1.4.3049, Scivis GmbH, Göttingen, Germany). SPECT and CT data were automatically co-registered and the fused datasets were analyzed with the VivoQuant post-processing software (version 2.50, inviCRO Imaging Services and Software, Boston, USA).

The mice were injected intravenously with ^47^Sc-DOTANOC (12 MBq, 1.2 nmol, 100 μL) and ^177^Lu-DOTANOC (40 MBq, 1.2 nmol, 100 μL), respectively. The in vivo SPECT/CT scans of 35 min duration were acquired 3 h after injection of ^47^Sc-DOTANOC. During the scans, the mice were anesthetized by inhalation of a mixture of isoflurane and oxygen. Post-mortem scans of 1.3–3.5 h were performed 6 h after injection of ^47^Sc- and ^177^Lu-DOTANOC. The SPECT acquisitions were performed in such a manner to obtain the same total number of counts for each scan.

## Results

### Production of ^47^Sc from ^47^Ti via the (n,p) reaction

The irradiation of enriched ^47^Ti targets at both SINQ and the BR2 reactor resulted in the formation of 0.07–4.9 MBq ^47^Sc at the end of irradiation (EOI). The respective ^47^Sc saturation yields were determined to be between 1.8 and 10.0 MBq ^47^Sc/mg ^47^Ti 10^−13^ n cm^−2^ s^−1^ (summarized in Table 2) by taking the irradiation time, mass of enriched ^47^Ti and fast neutron flux into consideration. γ-spectrometry measurements of the neutron-irradiated ^47^Ti ampoules revealed that, other than ^47^Sc, the long-lived radionuclidic impurity ^46^Sc was formed. The amount of generated ^46^Sc was influenced by the irradiation period and the neutron energy (Bokhari et al. 2010; Zerkin 2016) and ranged from 3.8 to 11.5% ^46^Sc/for the irradiations at SINQ (Additional file 1: Figure S3). Considerably less ^46^Sc (0.05%) was produced by the irradiation at the BR2 reactor, however. In view of the high percentage of co-produced ^46^Sc and the relatively low ^47^Sc production yield at both facilities, the production of sufficiently high ^47^Sc activities for radiopharmaceutical applications was not considered feasible and, thus, chemical isolation of ^47^Sc from neutron irradiated ^47^Ti targets was not performed.

**Table 2:** Activity and yield of ^47^Sc at the end of irradiation (EOI) with fast neutrons (>1 MeV) at SINQ (irradiations PSI 1, PSI 2 and PSI 3) and at the BR2 reactor (irradiation SCK.CEN)

Irradiation	t_irr_ [d]	m (^47^Ti) [mg]	A (^47^Sc) at EOI [MBq]	A (^47^Sc)_saturation_ [MBq/mg ^47^Ti 10^−13^ n cm^−2^ s^−1^]	^46^Sc activity at EOI [%]
PSI 1	10.9	19.03	3.9	6.6	7.8
PSI 2	18.9	15.11	4.9	10.0	11.5
PSI 3	1.5	1.31	0.07	6.9	3.8
SCK.CEN	7.0	0.58	4.7	1.8	0.05

### Production of ^47^Sc from ^46^Ca via the (n,γ) reaction

The irradiation of ^46^Ca targets with thermal neutrons resulted in the formation of ^47^Ca, which decayed to ^47^Sc and yielded 210–2140 MBq ^47^Sc at the time the separation was performed. The ^47^Sc saturation yield was determined by taking the mass of ^46^Ca, the irradiation time (t_irr_), the decay time after EOI (t_wait_) and the thermal neutron flux (Φ_th_) into account and was within the range of 85–98 MBq ^47^Sc/mg ^46^Ca 10^−13^ n cm^−2^ s^−1^, which is comparable with the calculated ^47^Sc saturation yield of 92 MBq ^47^Sc/mg ^46^Ca 10^−13^ n cm^−2^ s^−1^ (summarized in Table 3). ^47^Sc is formed during the irradiation but also, however, for some time after the end of irradiation by the decay of ^47^Ca. This implies that both the irradiation time (t_irr_ = 7.0–11.0 d) and the elapsed post-irradiation time until the start of the separation (t_wait_ = 3.0–12.5 d) are part of the yield-determining factors. The highest ^47^Sc activities under the applied irradiation conditions become accessible at an optimal post irradiation waiting time (t_opt_) and are represented as relative activity a(^47^Sc)_opt_ in Table 3. The measured relative ^47^Sc activities (a(^47^Sc)_meas_) are lower than the optimal relative ^47^Sc activities (a(^47^Sc)_opt_), as the separations were performed several days after the optimal waiting time. The variable f(^47^Sc) describes the ratio of the ^47^Sc activity at t_opt_ and the ^47^Ca activity at EOI. It can be considered as a measure for the maximal obtainable ^47^Sc activity, since both activities are referred to the time point of their maximum. The formulae used for the calculation of A(^47^Sc)_calc_, t_opt,_ a(^47^Sc)_opt_ and f(^47^Sc) are given in Additional file 1: Figure S1 a-d.

**Table 3:** Activity of ^47^Sc at the time of separation (A(^47^Sc)), comparison of the calculated and measured ^47^Sc saturation yield (A(^47^Sc)_calc_ and A(^47^Sc)_meas_) and of the optimal and measured relative ^47^Sc activity (a(^47^Sc)_opt_ and a(^47^Sc)_meas_) after irradiation with thermal neutrons at ILL (irradiations ILL 1–5) and BR2 (irradiation SCK.CEN)

Irradiation	t_irr_ [d]	t_wait_ [d]	t_opt_ [d]	m(^46^Ca) [mg]	A(^47^Sc) at separation [MBq]	A(^47^Sc)_calc_	A(^47^Sc)_meas_	a(^47^Sc)_opt_	a(^47^Sc)_meas_	f(^47^Sc)
						[MBq/mg ^46^Ca 10^−13^ n cm^−2^ s^−1^]				
ILL 1	7.0	6.7	2.8	0.17	690	92	86	0.43	0.34	0.65
ILL 2	7.2	6.7	2.7	0.35	1390	92	85	0.44	0.34	0.66
ILL 3	8.4	12.5	2.4	0.14	470	92	98	0.50	0.25	0.69
ILL 4	11.0	6.9	1.8	0.35	2140	92	95	0.62	0.49	0.76
ILL 5	7.0	6.8	2.8	0.35	1440	92	93	0.43	0.37	0.65
SCK.CEN	7.0	3.0	2.8	0.17	200	92	90	0.43	0.42	0.65

**Fig. 3:**
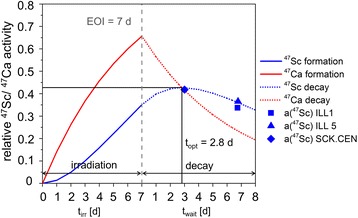
The relative ^47^Sc activities (a(^47^Sc)_meas_) of the irradiations ILL 1, ILL 5 and SCK.CEN accessible (and measured) at the time of separation (single data points). The activation functions of ^47^Sc and ^47^Ca at an irradiation period of 7.0 days (*solid* *blue* and *red line*) and their subsequent decay functions after the EOI (*dashed blue* and *red line*) are calculated for the respective irradiations

The measured relative ^47^Sc activities (a(^47^Sc)_meas_) of the irradiations ILL 1, ILL 5 and SCK.CEN are represented by single data points in Fig. 3. After an optimal post irradiation waiting time (t_opt_) of 2.8 days, the relative ^47^Sc activity (a(^47^Sc)_opt_) reaches the maximum with 0.43. The separations ILL 1 and ILL 5 were performed after waiting times of 6.7 and 6.8 days, resulting in lower relative ^47^Sc activities of 0.34 and 0.37, respectively. The waiting time after the irradiation at SCK.CEN (3.0 days) is close to t_opt_, thus the obtained relative ^47^Sc activity of 0.44 is comparable with the optimum value.

### Separation of ^47^Sc from ^46^Ca and ^47^Ca

After the ampoule was crushed, it was moved to the hot cell containing the production panel (Fig. 2), where the target material was dissolved by repeated application of 6.5 mL 3.0 M HCl. The ^47^Ca and ^47^Sc activity was transferred from the crushed glass ampoule to the DGA column, leaving only 1.1 ± 0.5% ^47^Ca and 3.3 ± 0.4% ^47^Sc attached to the glass. Direct application of 2 mL 3.0 M HCl quantitatively removed the Ca (^47^Ca and stable Ca isotopes) from the resin. The collected Ca fraction contained 99.8 ± 0.2% of the total ^47^Ca activity. The ^47^Sc activity was eluted from the DGA column with 4 mL 0.1 M HCl and only 1.5 ± 0.6% of the ^47^Sc activity remained on the column. Using Method A, the solution was concentrated on the SCX cation exchange resin (used as the second column) and eluted with 700 μL 4.8 M NaCl/0.1 M HCl solution (pH 0–0.5), collecting 94.8 ± 2.1% of the total ^47^Sc activity. When using Method B, the molarity of the ^47^Sc eluate was increased from 0.1 to 3.0 M HCl and the resulting solution adsorbed on a second smaller DGA column and eluted with 1.7 mL 0.05 M HCl. Fractionized collection revealed that about ~90% of the eluted ^47^Sc activity was obtained in the first 700 μL (pH ~0).

**Fig. 4:**
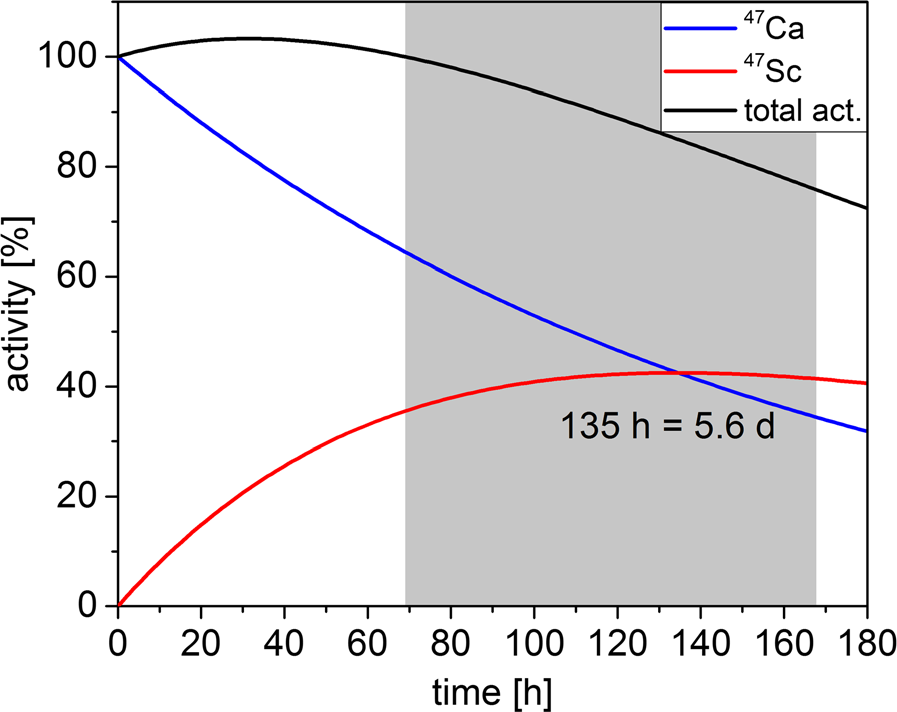
The radioactive decay of the parent nuclide ^47^Ca (T_1/2_ = 4.54 d) to the daughter nuclide ^47^Sc (T_1/2_ = 3.35 d) reaches the maximum of ^47^Sc activity after 135 h (5.6 d). The *grey-shaded* area indicates the time frame wherein the next separation was performed

With the installation of the chemical separation system in a hot cell, yields of up to 1.9 GBq ^47^Sc could be isolated from the irradiated ^46^Ca target. The renewed generation of ^47^Sc from the β^−^-decay of ^47^Ca (T_1/2_ = 4.54 d) in the Ca-containing fraction, reached the maximum ^47^Sc activity after an in-growth period of 5.6 days (Fig. 4) and, thus, enabled repeated separations. As a result of experimental conditions, separations were performed after an in-growth time of 3–7 days. The separation process was successfully repeated 2–4 times, until the eluted ^47^Sc activity was ~100 MBq.

### Radionuclidic purity of ^47^Sc produced from ^46^Ca via (n,γ) reaction

The γ-ray spectrum of the neutron-irradiated ^46^Ca target material (Additional file 1: Figure S2a) showed, exclusively, the γ-lines of ^47^Sc (159 keV) and the parent nuclide ^47^Ca (489, 808 and 1297 keV). After chemical separation and concentration of ^47^Sc on SCX resin (Method A), the radionuclidic purity of the final ^47^Sc eluate was 99.6 ± 0.7%. When the second DGA column (Method B) was used, the radionuclidic purity increased to 99.99 ± 0.03% (Additional file 1: Figure S2b). The long-lived radionuclidic impurity ^46^Sc was only present in the eluate obtained from the first separation at a maximum of 0.005% and could be only detected by performing long-term γ-spectrometry measurements several days post separation. The isolated ^47^Sc which was generated by the decay of ^47^Ca in the Ca-containing fraction did not contain any ^46^Sc, due to its entire removal within the first separation.

### Radiolabeling and stability of ^47^Sc labeled DOTANOC

Radiolabeling of ^47^Sc was reproducible at a specific activity of 10 MBq ^47^Sc per nmol DOTANOC, with >96% radiochemical purity. Depending on the activity concentration of the ^47^Sc solution, it was also possible to label at higher specific activity of up to 25 MBq/nmol.

**Fig. 5:**
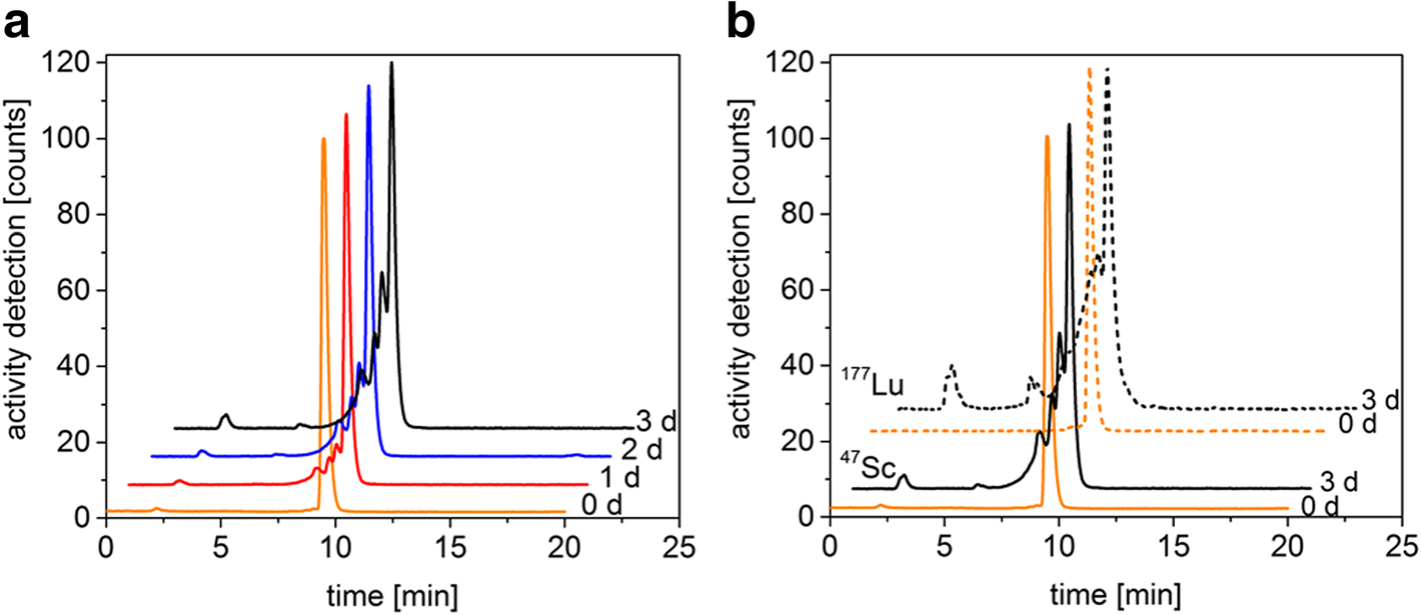
Stability of ^47^Sc-DOTANOC (**a**) and the comparison of the stability of ^47^Sc- and ^177^Lu-DOTANOC (**b**) in PBS (pH 7.4) investigated at room temperature over a 3-day period after radiolabeling. The retention times of free ^47^Sc and ^177^Lu are 2.2 ± 0.1 min and for ^47^Sc- and ^177^Lu-DOTANOC 9.5 ± 0.1 min and 9.3 ± 0.2 min, respectively

The stability of ^47^Sc-labeled DOTANOC in PBS (pH 7.4) was investigated over a period of 3 days and compared to the stability of the ^177^Lu-labeled analogue (Fig. 5). Directly after the radiosynthesis of ^47^Sc with DOTANOC, the amount of intact radiolabeled product was 96.6–99.0% with less than 2% of radiolysis products visible as pre-peaks on the HPLC chromatogram. After 1 day at room temperature, the amount of intact radiolabeled ^47^Sc-DOTANOC decreased to 81.3%, while 18.3% were subjected to radiolysis. Over the whole investigation period of 3 days, the percentage of radiolysis products increased to 44.4%, however, the amount of free ^47^Sc was always below 2.1%. The stability of ^47^Sc-labeled DOTANOC was found to be comparable with the clinically-used analogue ^177^Lu-DOTANOC. After 3 days, the amount of intact ^47^Sc-DOTANOC (54.1%) was similar to the amount of intact ^177^Lu-DOTANOC (43.2%).

### Imaging with ^47^Sc-DOTANOC in comparison to ^44^Sc-DOTANOC and ^177^Lu-DOTANOC

**Fig. 6:**
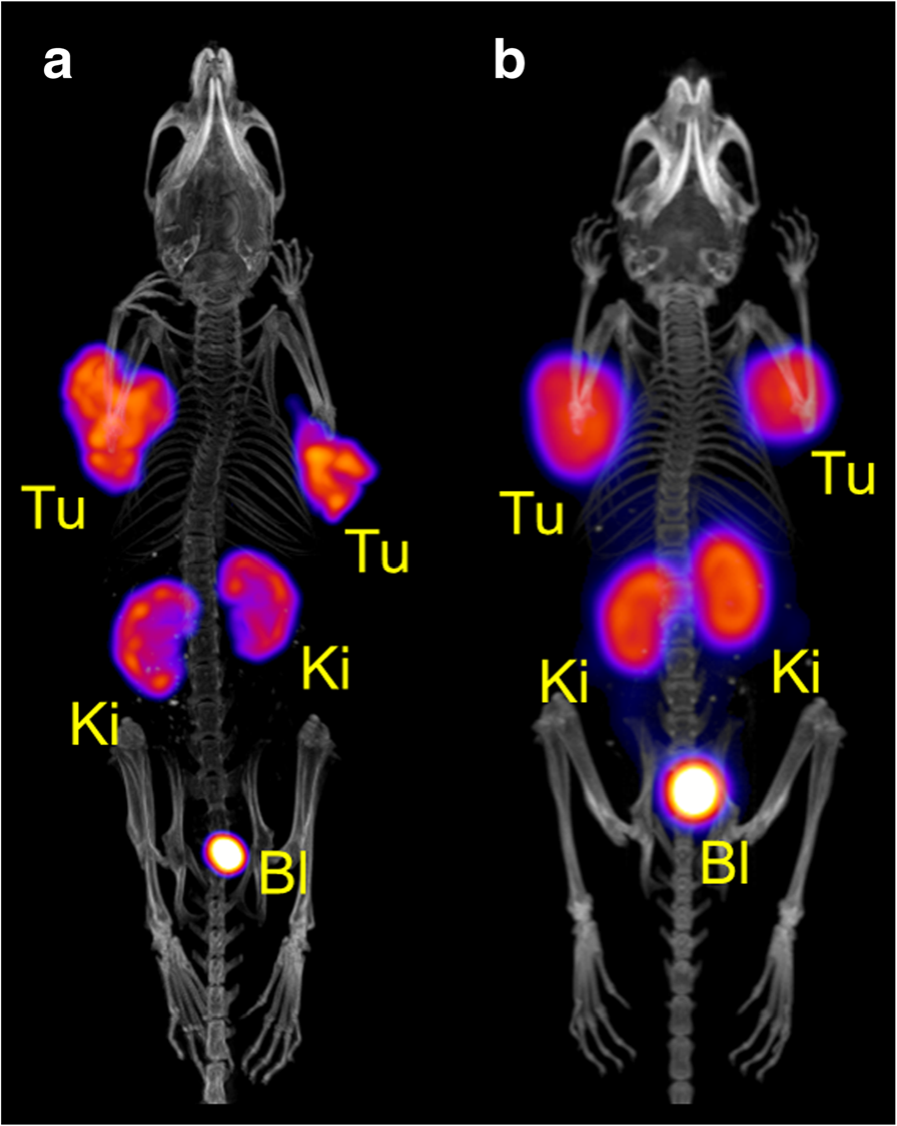
In vivo SPECT/CT scan of a tumor-bearing mouse 3 h after injection of ^47^Sc-DOTANOC (~12 MBq, ~1.2 nmol) (**a**). In vivo PET/CT scan of a tumor-bearing mouse 3 h after injection. ^44^Sc-DOTANOC (~10 MBq, ~1 nmol), image reproduced from Domnanich et al. 2016 (Domnanich et al. 2016) (**b**). The scan durations were 35 min and 20 min, respectively (Tu = AR42J tumor xenograft, Ki = kidney, Bl = urinary bladder)

SPECT/CT experiments performed with AR42J tumor-bearing mice allowed excellent visualization of the accumulated ^47^Sc-DOTANOC in tumor xenografts, which express the somatostatin receptor (Fig. 6a). Activity accumulation was also observed in the kidneys, which was due to renal excretion of the radiopeptide. The SPECT/CT image obtained with ^47^Sc-DOTANOC showed an equal activity distribution profile as was previously demonstrated by a PET/CT scan of an AR42J tumor-bearing mouse 3 h after injection of ^44^Sc-DOTANOC (Fig. 6b) (Domnanich et al. 2016).

**Fig. 7:**
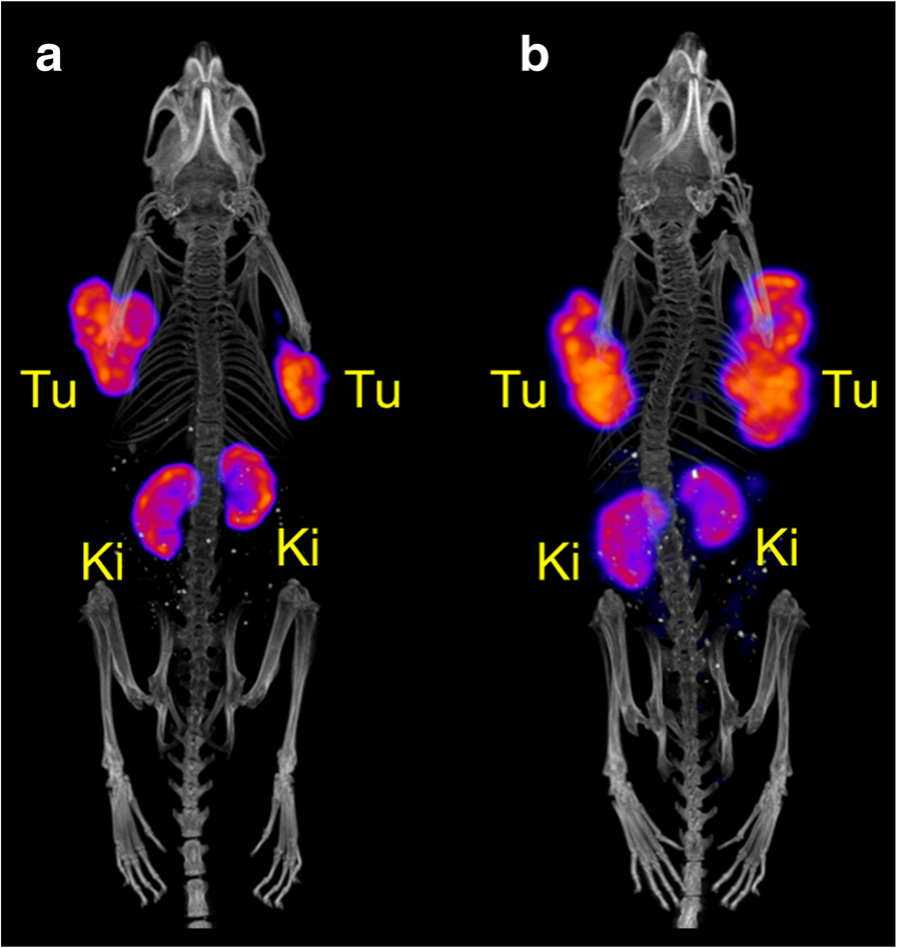
Post-mortem SPECT/CT scans of tumor-bearing mice 6 h after injection of the corresponding radiopeptide. Mouse injected with ^47^Sc-DOTANOC (~12 MBq, ~1.2 nmol) (**a**) and mouse injected with ^177^Lu-DOTANOC (~40 MBq, ~1.2 nmol) (**b**). The scan durations were 3.5 h and 1.3 h, respectively. (Tu = AR42J tumor xenograft, Ki = kidney)

To compare the image quality of ^47^Sc with the clinically-employed ^177^Lu, mice with AR42J tumors were injected with either ^47^Sc- or ^177^Lu-labeled DOTANOC and scanned 6 h after injection (Fig. 7). Both images visualized the uptake of the radiopeptides in tumor xenografts located on each shoulder of the mouse and in the kidneys. The distribution pattern was equal for both radiopeptides, as expected, based on similar coordination of ^47^Sc and ^177^Lu when using DOTA.

## Discussion

Recently, the use of ^47^Sc for therapeutic purposes, as part of the ^44^Sc/^47^Sc theragnostic radionuclide pair, has attracted considerable attention in the field of nuclear medicine (Rösch and Baum 2011). In the present study we report, to our knowledge, the first reproducible production of MBq to GBq activities of ^47^Sc by the irradiation of enriched ^46^Ca target material with thermal neutrons. Concurrently, an alternative production route was investigated, using ^47^Ti as target material and, by irradiation with fast neutrons, ^47^Sc was formed via the (n,p) nuclear reaction. Irradiation of enriched ^47^Ti at the spallation source SINQ (PSI, Switzerland) and the BR2 reactor (SCK.CEN, Mol, Belgium) resulted in the formation of 1.8–10.0 MBq ^47^Sc/mg ^47^Ti 10^−13^ n cm^−2^ s^−1^. The production of ^47^Sc by neutron irradiation of enriched ^47^Ti target material was performed previously by Mausner and Kolsky in the fission neutron spectrum of the HFIR reactor in Oak Ridge National Laboratory (ORNL) and Brookhaven National Laboratory (BNL) (Kolsky et al. 1998, Mausner, Kolsky et al. 1998) and by Bartoś et al. at the Maria reactor in Świerk, Poland (Bartoś et al. 2012). The reported activity of produced ^47^Sc was within the range of 1.4–4.2 MBq ^47^Sc/mg ^47^Ti 10^−13^ n cm^−2^ s^−1^, which is comparable with the ^47^Sc radioactivity obtained in our experiment at BR2. The irradiations at SINQ generated higher ^47^Sc activities, due to the larger proportion of fast neutrons (> 1 MeV) at the spallation source (Lehmann 2016) than at the reactor (Chrysanthopoulou et al. 2014). The production of 85–98 MBq ^47^Sc/mg ^46^Ca 10^−13^ n cm^−2^ s^−1^ was feasible via the ^46^Ca(n,γ)^47^Ca → ^47^Sc route with thermal neutrons from nuclear reactors at ILL and BR2, however. The nearly ten-fold higher accessible ^47^Sc activity from ^46^Ca irradiations can be attributed to the higher nuclear cross section of the ^46^Ca(n,γ)^47^Ca reaction (σ = 0.7 barn) (Magill et al. 2015) in comparison to the ^47^Ti(n,p)^47^Sc nuclear reaction (Additional file 1: Figure S4), as well as to an increased flux of thermal neutrons in comparison to fast neutrons. Lower measured relative ^47^Sc activities than the optimal activities were obtained for the irradiations ILL 1–ILL 5, because the post-irradiation waiting period was too long and the built up ^47^Sc already started to decay. Shorter waiting periods were prevented due to logistical issues. The waiting period after the irradiation at SCK.CEN, however, was close to the optimum time period, thus the measured relative ^47^Sc activity obtained from the irradiation at SCK.CEN is in good correlation with the optimal value.

γ-spectra of the irradiated ^46^Ca indicated the presence of ^47^Ca and ^47^Sc, however, due to high dead time a precise determination of both activities before separation was not possible. After chemical separation a product of high radionuclidic purity, containing only 0.005% ^46^Sc, was obtained (Additional file 1: Figure S2). The acquired radionuclide inventory of ^47^Ti targets, neutron irradiated at the spallation source SINQ, indicated a higher percentage of ^46^Sc (Additional file 1: Figure S3), which increased proportionally with irradiation time (3.8–11.5% ^46^Sc for 1.5–18.9 days irradiation), whereas a significantly smaller amount of ^46^Sc (0.05%) was produced after 7 days of irradiation at the BR2 reactor (SCK.CEN). Trace activities of ^122^Sb and ^124^Sb were identified in the γ-ray spectrum of the irradiated ^47^Ti ampoule at SINQ (Additional file 1: Figure S3), conceivably produced by neutron activation reactions on natural Sb impurities (Additional file 1: Table S5). The presence of ^22^Na and ^7^Be can be attributed to spallation reactions with the capsulation material. The scan for trace metals revealed impurities of Ca, Sr, Sb and Zr in the reduced ^47^Ti metal (Additional file 1: Table S5), which were probably introduced by the reduction process. Neutron activation reactions were only observed with Sb, but not with any of the other determined impurities, however.

The formation of ^46^Sc from ^47^Ti via the ^47^Ti(n,n + p)^46^Sc nuclear reaction is known to be only induced by very fast neutrons above the threshold of 10.7 MeV (Additional file 1: Figure S4) (Zerkin 2016). The considerably decreased ^46^Sc impurity of the sample irradiated at the BR2 reactor can, therefore, be attributed to the lower proportion of very energetic neutrons in the fission spectrum of the BR2 reactor (Chrysanthopoulou et al. 2014), compared to the spallation neutron spectrum in the SINQ (Lehmann 2016). The percentage of ^46^Sc obtained from the ^47^Ti irradiation at the BR2 reactor was in agreement with those from previous experiments at ORNL, BNL and the Maria reactor, which was reported to be 0.06–0.64% ^46^Sc (Kolsky et al. 1998; Mausner et al. 1998; Bartoś et al. 2012). With respect to radiopharmaceutical applications, the ten-fold higher ^47^Sc production from ^46^Ca targets, together with the absence of long-lived radionuclidic impurities, intensified our research towards the further development of the more attractive ^46^Ca route.

In order to meet the requirements for radiopharmaceutical applications, the obtained ^47^Sc eluate needed to be of high chemical purity and concentrated into a small volume of moderately acidic eluate to facilitate efficient radiolabeling and subsequent in vivo application. Initially, SCX cation exchange resin (Method A) was used and 94.8% ± 2.1% of the total ^47^Sc activity was recovered in only 700 μL eluate (4.8 M NaCl/0.1 M HCl). The use of this resin is already established for the concentration of the ^68^Ga eluate from the ^68^Ge generator (Mueller et al. 2013); however, direct preclinical in vivo application is not feasible due to the high osmolarity of the obtained eluate. In a modification of the separation procedure, a second, smaller DGA column was used (Method B), allowing the elution of ~90% using 700 μL 0.05 M HCl. This enabled labeling and preclinical application as previously shown with ^44^Sc (Domnanich et al. 2016).

The labeling of DOTANOC with ^47^Sc was performed to verify a consistent chemical purity of the obtained eluate. Our results demonstrated reproducible radiosynthesis of ^47^Sc-DOTANOC at specific activities of 10 MBq/nmol, whereas radiolabeling at 25 MBq/nmol proved possible. The obtained results indicated good quality of the produced ^47^Sc achieving radiolabeling yields, in agreement with the previously-performed ^47^Sc-radiolabeling of a DOTA-folate conjugate (Müller et al. 2014a).

In PBS ^47^Sc remained stably coordinated by the DOTA-chelator over 3 days (<6% release), a result which was comparable to the ^177^Lu-labeled peptide. ^47^Sc- and ^177^Lu-DOTANOC were, however, affected by radiolytic decomposition, which decreased the amount of intact product over time. It is likely the radiolytic stability could be enhanced by the addition of radical scavengers, such as ascorbic or gentisic acids, which were previously successfully employed for the stabilization of ^90^Y- and ^177^Lu-labeled DOTA-peptides (Liu and Edwards 2001; Liu et al. 2003).

In a proof-of-concept study, ^47^Sc-DOTANOC was utilized for SPECT/CT imaging of AR42J tumor-bearing mice. The equal distribution profile of ^47^Sc-DOTANOC and ^44^Sc-DOTANOC, previously demonstrated using PET/CT, demonstrated the successful realization of the “matched pair” principle using scandium radionuclides. Moreover, the in vivo distribution of ^47^Sc-DOTANOC was comparable to ^177^Lu-DOTANOC. Due to the higher percentage of emitted γ-radiation in the case of ^47^Sc, it is expected that less activity of ^47^Sc-labeled compounds would be necessary for clinical SPECT as compared to the activity necessary for ^177^Lu-labeled counterparts.

## Conclusions

The reproducible production of activities of up to 2 GBq ^47^Sc at high radionuclidic purity via the ^46^Ca(n,γ)^47^Ca nuclear reaction in the thermal neutron flux of a reactor was demonstrated. The subsequent decay of ^47^Ca to ^47^Sc creates a “pseudo-generator” system, which enables the repeated separation of the ^47^Sc daughter nuclide from the ^47^Ca parent nuclide. Together with the high radionuclidic purity and the superior yield of the isolated ^47^Sc activity, the ^46^Ca production route bears significant advantages over the ^47^Ti production route with fast neutrons. Even though the high price of enriched ^46^Ca represents a drawback, implementation of a suitable recovery method will limit the expenses. Based on the results obtained from this proof-of-concept study, ^47^Sc has the potential to be produced in a suitable quality for clinical applications, however, the quantity of radioactivity still needs to be expanded to meet the requirements for radionuclide therapy.

## Additional file

Additional file 1: Figure S1a. Formula for the calculation of the ^47^Sc activity in Bq (s^−1^), accessible under the applied irradiation conditions. σ = nuclear cross section of the ^46^Ca(n,γ)^47^Ca reaction in cm^−2^, N_T_ = number of ^46^Ca atoms, Φ_th_ = thermal neutron flux in n * cm^−2^ * s^−1^, λ_Sc_ and λ_Ca_ = decay constants of ^47^Sc and ^47^Ca in s^−1^, t_irr_ = irradiation time and t_wait_ = post irradiation waiting time in s. **b** Formula for the calculation of the optimal post irradiation waiting time (t_opt_) in s, accessible at the applied irradiation time (t_irr_ in s). The decay constants of ^47^Sc (λ_Sc_) and ^47^Ca (λ_Ca_) are given in s^−1^. **c** Formula for the calculation of the optimal relative ^47^Sc activity (a(^47^Sc)_opt_) (dimensionless), accessible under the applied irradiation conditions. σ = nuclear cross section of the ^46^Ca(n,γ)^47^Ca reaction in cm^−2^, N_T_ = number of ^46^Ca atoms, Φ_th_ = thermal neutron flux in n * cm^−2^ * s^−1^, λ_Sc_ and λ_Ca_ = decay constants of ^47^Sc and ^47^Ca in s^−1^. **d** Formula for the maximal obtainable ^47^Sc activity (dimensionless). The irradiation time (t_irr_) is given in s and the decay constants of ^47^Sc (λ_Sc_) and ^47^Ca (λ_Ca_) in s^−1^. **Figure S2.** γ-Ray spectra of ^47^Sc and ^47^Ca from the neutron-irradiated ^46^Ca ampoule, obtained 71 h after the end of irradiation (measurement time: 10 s) (**a**) and of the pure ^47^Sc eluate after separation (Method B), obtained 1 h after the end of separation (measurement time: 250 s) (**b**). **Figure S3.** γ-Ray spectrum of the neutron-irradiated ^47^Ti ampoule at SINQ, obtained 21 d after the end of irradiation (measurement time: 9600 s). **Figure S4.** Measured cross section values (squares, retrieved from the EXFOR-database) (Zerkin 2016) as well as the theoretical calculations from the TENDL-2015 library (straight line) (Koning, Rochman et al. 2015) for the ^47^Ti(n,p)^47^Sc (blue) and the ^47^Ti(n,p + n)^46^Sc (black) nuclear reactions. **Table S5.** Trace metal analysis of the reduced ^46^Ti metal by ICP-OES. Only the elements determined at a concentration higher than the detection limit are listed below. (DOCX 415 kb)
