# Supplementary material for: 47Sc as useful β–-emitter for the radiotheragnostic paradigm: a comparative study of feasible production routes
Source: EJNMMI Radiopharm Chem. 2017 Jun 2;2:5. doi: 10.1186/s41181-017-0024-x (PMC5824697; doi:10.1186/s41181-017-0024-x)
Supplement: Supplementary file 1 — Formula for the calculation of the 47Sc activity in Bq (s−1), accessible under the applied irradiation conditions. σ = nuclear cross section of the 46Ca(n,γ)47Ca reaction in cm−2, NT = number of 46Ca atoms, Φth = thermal neutron flux in n * cm−2 * s−1, λSc and λCa = decay constants of 47Sc and 47Ca in s−1, tirr = irradiation time and twait = post irradiation waiting time in s. b Formula for the calculation of the optimal post irradiation waiting time (topt) in s, accessible at the applied irradiation time (tirr in s). The decay constants of 47Sc (λSc) and 47Ca (λCa) are given in s−1. c Formula for the calculation of the optimal relative 47Sc activity (a(47Sc)opt) (dimensionless), accessible under the applied irradiation conditions. σ = nuclear cross section of the 46Ca(n,γ)47Ca reaction in cm−2, NT = number of 46Ca atoms, Φth = thermal neutron flux in n * cm−2 * s−1, λSc and λCa = decay constants of 47Sc and 47Ca in s−1. d Formula for the maximal obtainable 47Sc activity (dimensionless). The irradiation time (tirr) is given in s and the decay constants of 47Sc (λSc) and 47Ca (λCa) in s−1. Figure S2. γ-Ray spectra of 47Sc and 47Ca from the neutron-irradiated 46Ca ampoule, obtained 71 h after the end of irradiation (measurement time: 10 s) (a) and of the pure 47Sc eluate after separation (Method B), obtained 1 h after the end of separation (measurement time: 250 s) (b). Figure S3. γ-Ray spectrum of the neutron-irradiated 47Ti ampoule at SINQ, obtained 21 d after the end of irradiation (measurement time: 9600 s). Figure S4. Measured cross section values (squares, retrieved from the EXFOR-database) (Zerkin 2016) as well as the theoretical calculations from the TENDL-2015 library (straight line) (Koning, Rochman et al. 2015) for the 47Ti(n,p)47Sc (blue) and the 47Ti(n,p + n)46Sc (black) nuclear reactions. Table S5. Trace metal analysis of the reduced 46Ti metal by ICP-OES. Only the elements determined at a concentration higher than the detection limit are listed below. ( [file 41181_2017_24_MOESM1_ESM.docx]

Supplementary Material

**^47^Sc as useful β^-^-emitter for the radiotheragnostic paradigm: a comparative study of feasible production routes**

Katharina A. Domnanich^1,2^, Cristina Müller^3,4^, Martina Benešová^3,4^, Rugard Dressler^1^, Stephanie Haller^3^, Ulli Köster^5^, Bernard Ponsard^6^, Roger Schibli^3,4^, Andreas Türler^1,2^, Nicholas P. van der Meulen^1,3*^

*^1^Laboratory of Radiochemistry, Paul Scherrer Institut, 5232 Villigen-PSI, Switzerland*

*^2^Department of Chemistry and Biochemistry University of Bern, 3012 Bern, Switzerland*

*^3^Center for Radiopharmaceutical Sciences ETH-PSI-USZ, Paul Scherrer Institut, 5232 Villigen-PSI, Switzerland*

*^4^Department of Chemistry and Applied Biosciences, ETH Zurich, 8093 Zurich, Switzerland*

*^5^Institut Laue-Langevin, 38000 Grenoble, France*

*^6^SCK.CEN, BR2 Reactor, 2400 Mol, Belgium*

E-mail addresses:

[katharina.domnanich@psi.ch](mailto:katharina.domnanich@psi.ch); [cristina.mueller@psi.ch](mailto:cristina.mueller@psi.ch); [martina.benesova@psi.ch](mailto:martina.benesova@psi.ch); [steffihaller@gmx.ch](mailto:steffihaller@gmx.ch); [rugard.dressler@psi.ch](mailto:rugard.dressler@psi.ch); [koester@ill.fr](mailto:koester@ill.fr); [bernard.ponsard@sckcen.be](mailto:bernard.ponsard@sckcen.be); [roger.schibli@psi.ch](mailto:roger.schibli@psi.ch); [andreas.tuerler@psi.ch](mailto:andreas.tuerler@psi.ch); [nick.vandermeulen@psi.ch](mailto:nick.vandermeulen@psi.ch)

*** Corresponding author:**

Dr. Nicholas P. van der Meulen

Laboratory of Radiochemistry and Center for Radiopharmaceutical Science ETH/PSI/USZ

Paul Scherrer Institut

5232 Villigen-PSI

Switzerland

e-mail: nick.vandermeulen@psi.ch

phone: +41-56-310 50 87

fax: +41-56-310 28 49

$$A({}^{47}{Sc})= \sigma*N_{T}* \Phi_{th}* \left[ \frac{\lambda_{Sc}\left( 1-e^{{-\lambda}_{Ca}*t_{irr}} \right)*e^{{-\lambda}_{Ca}*t_{wait}}-\lambda_{Ca}\left( 1-e^{{-\lambda}_{Sc}*t_{irr}} \right)*e^{{-\lambda}_{Sc}*t_{wait}}}{\lambda_{Sc}-\lambda_{Ca}} \right]$$

**Figure S1 a** Formula for the calculation of the ^47^Sc activity in Bq (s^-1^), accessible under the applied irradiation conditions. σ = nuclear cross section of the ^46^Ca(n,γ)^47^Ca reaction in cm^-2^, N_T_ = number of ^46^Ca atoms, Φ_th_ = thermal neutron flux in n * cm^-2^ * s^-1^, λ_Sc_ and λ_Ca_ = decay constants of ^47^Sc and ^47^Ca in s^-1^, t_irr_ = irradiation time and t_wait_ = post irradiation waiting time in s.

$$t_{opt}=\frac{1}{\lambda_{Sc}-\lambda_{Ca}}*ln\left[ \frac{\left( 1-e^{{-\lambda}_{Sc}*t_{irr}} \right)}{\left( 1-e^{{-\lambda}_{Ca}*t_{irr}} \right)} \right]$$

**Figure S1 b** Formula for the calculation of the optimal post irradiation waiting time (t_opt_) in s, accessible at the applied irradiation time (t_irr_ in s). The decay constants of ^47^Sc (λ_Sc_) and ^47^Ca (λ_Ca_) are given in s^-1^.

$$a{({}^{47}{Sc)}}_{opt}=\frac{{(1-e^{-\lambda_{Ca}*t_{irr}})}^{\frac{\lambda_{Sc}}{\lambda_{Sc}-\lambda_{Ca}}}}{{(1-e^{-\lambda_{Sc}*t_{irr}})}^{\frac{\lambda_{Ca}}{\lambda_{Sc}-\lambda_{Ca}}}}$$

**Figure S1 c** Formula for the calculation of the optimal relative ^47^Sc activity (a(^47^Sc)_opt_) (dimensionless), accessible under the applied irradiation conditions. σ = nuclear cross section of the ^46^Ca(n,γ)^47^Ca reaction in cm^-2^, N_T_ = number of ^46^Ca atoms, Φ_th_ = thermal neutron flux in n * cm^-2^ * s^-1^, λ_Sc_ and λ_Ca_ = decay constants of ^47^Sc and ^47^Ca in s^-1^.

$$f({}^{47}{Sc)=\frac{{A({}^{47}{Sc)}}_{opt}}{{A({}^{47}{Ca)}}_{EOI}}{=\left[ \frac{1-e^{-\lambda_{Ca}*t_{irr}}}{1-e^{-\lambda_{Sc}*t_{irr}}} \right]}^{\frac{\lambda_{Ca}}{\lambda_{Sc}-\lambda_{Ca}}}}$$

**Figure S1 d** Formula for the maximal obtainable ^47^Sc activity (dimensionless). The irradiation time (t_irr_) is given in s and the decay constants of ^47^Sc (λ_Sc_) and ^47^Ca (λ_Ca_) in s^-1^.


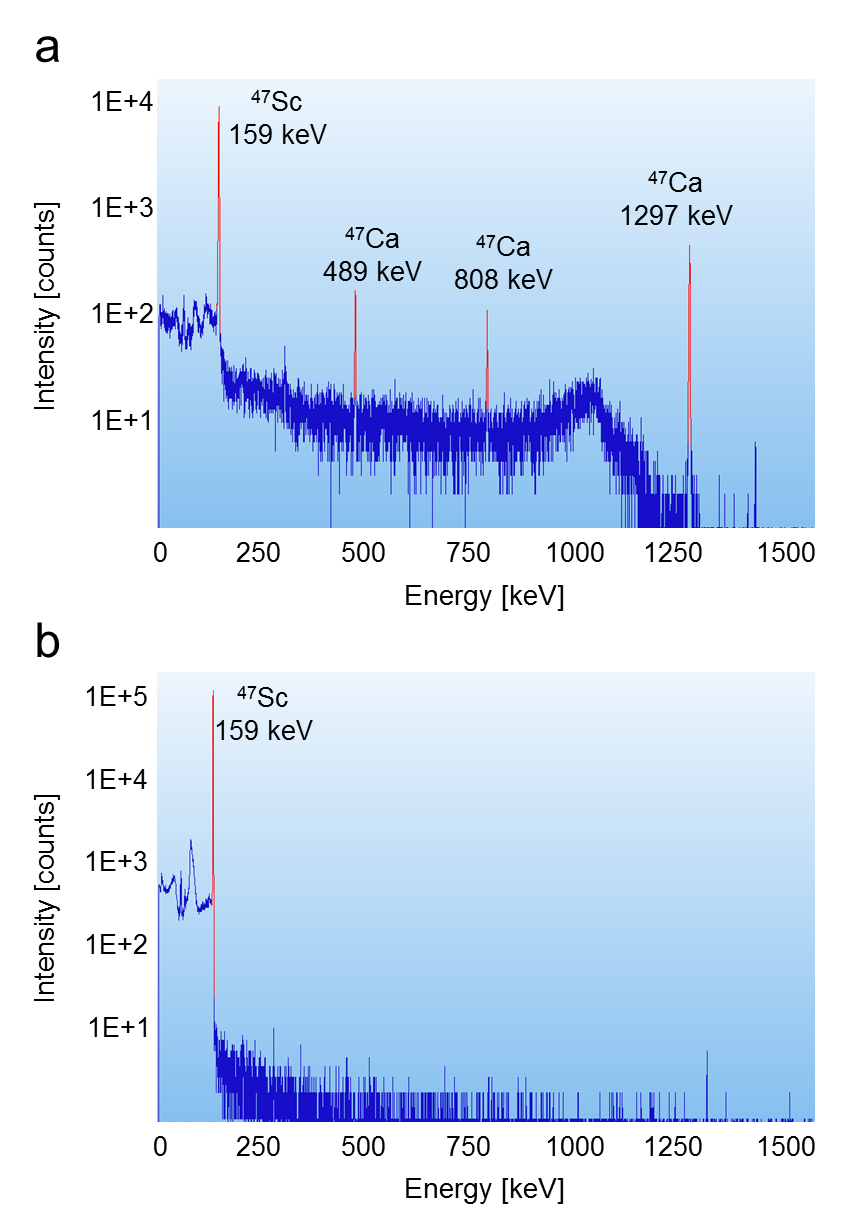


**Figure S2** γ-Ray spectra of ^47^Sc and ^47^Ca from the neutron-irradiated ^46^Ca ampoule, obtained 71 h after the end of irradiation (measurement time: 10 s) (**a**) and of the pure ^47^Sc eluate after separation (Method B), obtained 1 h after the end of separation (measurement time: 250 s) (**b**).

**
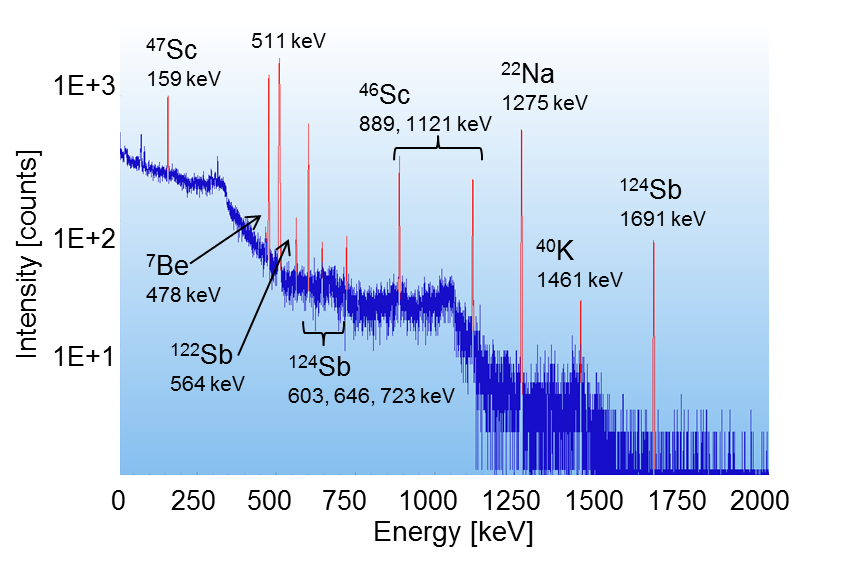
**

**Figure S3:** γ-Ray spectrum of the neutron-irradiated ^47^Ti ampoule at SINQ, obtained 21 d after the end of irradiation (measurement time: 9600 s).


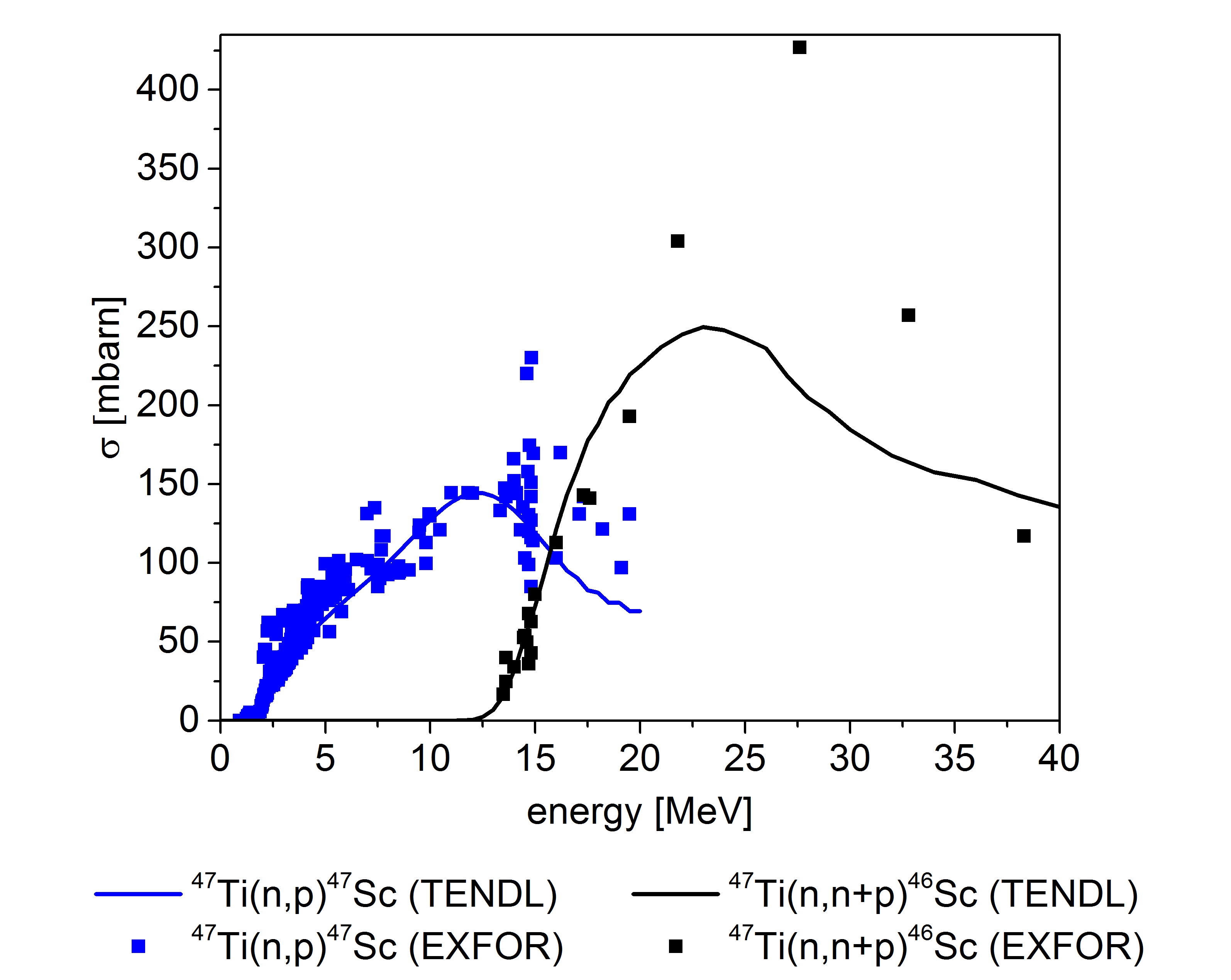


**Figure S4** Measured cross section values (squares, retrieved from the EXFOR-database) (Zerkin 2016) as well as the theoretical calculations from the TENDL-2015 library (straight line) (Koning, Rochman et al. 2015) for the ^47^Ti(n,p)^47^Sc (blue) and the ^47^Ti(n,p+n)^46^Sc (black) nuclear reactions.

**Table S5** Trace metal analysis of the reduced ^46^Ti metal by ICP-OES. Only the elements determined at a concentration higher than the detection limit are listed below.

| Element | Ratio of element per mg of Ti |
| --- | --- |
| Ca | 1.4*10^-2^ |
| Sr | 8.5*10^-4^ |
| Sb | 2.2*10^-4^ |
| Ti | 1 |
| Zr | 9.9*10^-4^ |

**References**

Koning, A. J., D. Rochman, J. Kopecky, C. Sublet, E. Bauge, S. Hilaire, P. Romain, B. Morillon, H. Duarte, S. van der Marck, S. Pomp, H. Sjostrand, R. Forrest, H. Henriksson, O. Cabellos, S. Goriely, J. Leppanen, H. Leeb, A. Plompen and R. Mills. (2015). "TENDL-2015: TALYS-based evaluated nuclear data library." Retrieved 05.12., 2016, from https://tendl.web.psi.ch/tendl_2015/tendl2015.html.

Zerkin, V. (2016). "Experimental Nuclear Reaction Data (EXFOR)." Retrieved 05.12., 2016, from https://www-nds.iaea.org/exfor/exfor.htm.
